# Evaluation of the Effects of Preoperative Dexamethasone Administration on Postoperative Patient Comfort in Laparoscopic Cholecystectomy

**DOI:** 10.7759/cureus.7968

**Published:** 2020-05-05

**Authors:** Gungor Gul, Tayfun Bilgic, Mehmet Akif Aydin

**Affiliations:** 1 General Surgery, Private Goztepe Hospital, Istanbul, TUR; 2 Medical Service and Techniques, Nisantasi University Vocational High School, Istanbul, TUR; 3 General Surgery, Altinbas University Faculty of Medicine, Medical Park Bahcelievler Hospital, Istanbul, TUR

**Keywords:** gallstones, laparoscopic cholecystectomy, dexamethasone, pain, nausea, vomiting

## Abstract

Objective

Postoperative recovery process following laparoscopic cholecystectomy depends on many factors such as pain, fatigue and exhaustion. The objective of this study was to investigate whether the administration of dexamethasone, a glucocorticoid, has positive effects of postoperative patient comfort in patients who underwent laparoscopic cholecystectomy in our clinic.

Methods

Patients who presented to the general surgery clinic of our hospital and scheduled for laparoscopic cholecystectomy due to cholelithiasis were included in this study. Patients in Group 1 received dexamethasone 90 minutes before the skin incision, while patients in Group 2 were given placebo (normal saline). Pain scores, presence of nausea and vomiting in the postoperative period were compared between the study and control groups.

Results

No statistically significant difference was observed between the groups in terms of incisional pain at rest and in motion and visceral pain at rest at postoperative 6th, 12th and 24th hours. Although there was a difference between the groups in terms of visceral pain in motion at the postoperative 12th and 24th hours, this was not statistically significant (p > 0.05). Although the need for additional analgesics and antiemetic drugs was lower in the study group compared to the control group, the difference between the groups was not statistically significant (p > 0.05).

Conclusion

We can expect better results with the use of multimodal analgesic and anti-emetic combination instead of a single agent in studies to be performed about the prevention of postoperative pain, nausea and vomiting.

## Introduction

Gallstone disease is a commonly seen problem in developed countries. Autopsy and clinical investigations have shown that at least 10% of adults have gallstones. Whereas 40-60% of people with gallstones exhibit a symptomatic clinical course, the majority of patients with symptomatic cholelithiasis have an asymptomatic period. Twenty percent of symptomatic patients with gallstones present with acute cholecystitis picture, while 10% present with complicated cholecystitis (jaundice, cholangitis, pancreatitis), and 60-70% with chronic cholecystitis symptoms [1].

Surgery is a trauma applied on a patient. The organism produces a metabolic and endocrine response against trauma as a result of the stimulation of hypothalamus-pituitary-adrenal axis and sympathetic nervous system [2]. Studies have shown that response given to trauma is proportional to the trauma severity. Therefore, minimizing trauma is one of the main goals during surgical interventions [2, 3].

Recently, a golden page was opened in gallstone disease with the introduction of endoscopy in surgical operations and laparoscopically performed cholecystectomy. Laparoscopy is attractive due to several reasons: laparoscopic cholecystectomy is more cosmetic, postoperative pain is less, length of stay in hospital is shorter and return to normal activity and work is earlier with laparoscopic cholecystectomy [4].

Postoperative recovery process following laparoscopic cholecystectomy depends on many factors such as pain, fatigue and exhaustion. Pain and exhaustion are marked especially on the day of operation and next day, while nausea and vomiting mostly occur on the day of surgery, and rarely prolong postoperative recovery process.

Various agents are used in the management of postoperative process. Among these, glucocorticoids, although their mechanism of action has not yet been fully clarified, are known for their analgesic, antiinflammatory, antiemetic and immunomodulatory effects. Dexamethasone is a synthetic glucocorticoid used for its analgesic and antiinflammatory effects. In addition to these indications, this agent is also recommended for nausea and vomiting treatment which may occur during the postoperative process.

The objective of this study was to investigate whether preoperative dexamethasone administration will have positive effects of postoperative patient comfort in patients who underwent laparoscopic cholecystectomy in our clinic.

## Materials and methods

Patients who presented to the general surgery clinic of our hospital and scheduled for laparoscopic cholecystectomy due to cholelithiasis were included in this study. Patients with an American Society of Anesthesiologists (ASA) score of III or IV, those aged over 75 years, patients who underwent papillotomy with endoscopic retrograde cholangiopancreatography (ERCP) one month before the operation or later and pregnant women were excluded from the study. In addition, patients with additional diseases other than cholelithiasis that may cause pain, those with hepatic, renal, endocrine and immunologic diseases, patients who used opioids or tranquilizer for longer than one week before the operation and those with alcohol and/or substance abuse were also excluded.

Patients were randomly divided into two groups with computer generated random numbers. Patients in Group 1 received dexamethasone (8 mg, Dekort Amp 2 mL; DEVA ILAC) 90 minutes before the skin incision, while patients in Group 2 were given placebo (normal saline). The patients, surgeon and anesthetists were blind to the drugs administered.

Similar drugs were used in the anesthesia process of all patients. Vital findings of the patients were monitored in the postoperative recovery room. All patients were administered intramuscular nonsteroidal anti-inflammatory drugs (NSAID) and intravenous metoclopramide HCL in the postoperative recovery room at the postoperative 4th hour. Oral paracetamol tablet was initiated after the postoperative 8th hour and patients were advised to use it regardless of the presence of pain. Other additional analgesic and anti-emetic drugs were systematically recorded for the first 24 hours. Recovery time was defined as the time from wound closure to the decision of taking patients to the ward following vital findings in the postanesthesia care unit.

Pain was daily recorded at the postoperative 6th, 12th and 24th hours and postoperative 1st week. Incisional pain was defined as superficial pain localized in the wound site or abdominal pain. Visceral pain was considered as blunt intraabdominal pain, which was difficult to locate. These pains were separately recorded as at rest and in motion at above specified periods of time. Total pain was evaluated as the sum of incisional and visceral pains at rest and in motion beginning from the postoperative first day for one week. Visual analogue scale (VAS) was used for pain scoring. Patients were asked to choose the most appropriate number describing their condition for a scale involving numbers between 1 and 10 (1: no pain, 10: unbearable pain) [5,6].

Nausea and vomiting periods were evaluated at two distinct intervals as postop 0-6 hours and 6-12 hours. Nausea was evaluated with verbal rating scale (VRS) (0: no nausea, 1: mild nausea, 2: moderate nausea, 3: severe nausea). Vomiting was recorded according to the number of vomiting (0: no vomiting, 1: vomiting once, 2: vomiting 2-3 times, 3: vomiting more than 3 times) [5-7].

Ethics statement

Before the beginning, the study protocol was approved by the local ethics committee of our hospital. Patients were informed about the objectives of the study and possible side effects in details and gave written consent. The study was conducted in accordance with the ethical principles of the Declaration of Helsinki.

Statistical analysis

Data obtained in the study were statistically analyzed using SPSS for Windows version 10.0 statistical package software. Besides descriptive statistical methods (mean, standard deviation), in the comparison of quantitative variables, student t test was used in the comparison of normally distributed data between the study and control groups, and Mann-Whitney U test in the comparison of non-normally distributed data. Qualitative variables were compared between the groups with Chi-square test. p < 0.05 values were considered statistically significant.

## Results

The study was conducted in the general surgery clinic of our hospital with 60 patients. Of all patients, 43 (71.7%) were female and 17 (28.3%) male. Patients were divided into two groups as the study and control groups with 30 patients in each. The mean age of the patients was 46.13 ± 14.12 (min-max: 20-71 years).

No statistically significant difference was found between the groups in terms of age and height (p > 0.05). No significant difference was observed between both groups in terms of gender (p > 0.05). The number of female patients was higher than male patients in both groups. There was no statistically significant difference between the groups in terms of ASA scores (p > 0.05). Majority of patients had an ASA score of I in both groups. Demographic data of the groups are given in Table 1.

**Table 1:** Demographic features of the groups ASA: American Society of Anesthesiologists

	Study Group	Control Group	p
	Median (min-max)	Median (min-max)	
Age (year)	47 (20-69)	45 (22-70)	0.959
Weight (kg)	70 (54-98)	68 (51-87)	0.277
Gender	n (%)	n (%)	
Female	21 (67.7)	22 (75.9)	0.485
Male	10 (32.3)	7 (24.1)	
ASA			
I	24 (77.4)	20 (69.0)	0.459
II	7 (22.6)	9 (31.0)	

There was no statistically significant difference between the groups in terms of anesthesia duration and operation time (both p > 0.05). Recovery time was statistically significantly lower in the study group compared to the control group (p < 0.05). No statistically significant difference was found between both groups in terms of the duration of hospitalization (p > 0.05). Clinical features of the groups are shown in Table 2.

**Table 2:** Clinical features of the groups SD: Standard deviation

	Study Group	Control Group	p
	Mean + SD	Mean + SD	
Anesthesia duration (min)	72 + 11.9	66.1 + 18.3	0.140
Operation time (min)	54.4 + 10.1	52.2 + 15.7	0.523
Recovery time (min)	34.0 + 7.9	38.3 + 7.8	0.041
Hospitalization (day)	1.2 + 0.4	1.2 + 0.4	0.660
Postop nausea vomiting	n (%)	n (%)	
Yes	6 (19.4)	5 (17.2)	0.485
No	25 (80.6)	24 (82.8)	0.833

No statistically significant difference was observed between the group in terms of incisional pain at rest at postoperative 6th, 12th and 24th hours. Again, no statistically significant difference was observed between the group in terms of incisional pain in motion at postoperative 6th, 12th and 24th hours.

**Figure 1:**
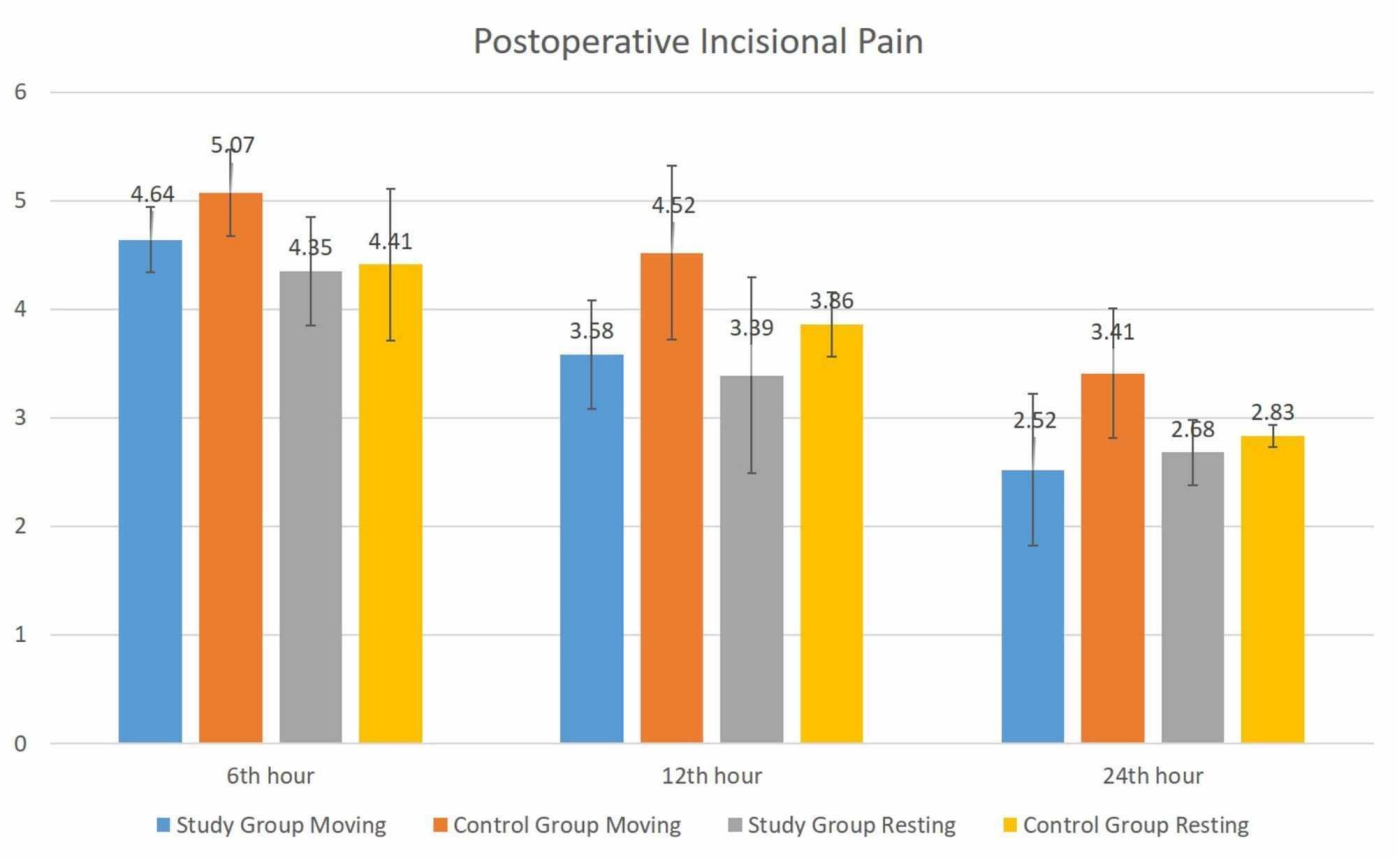
Postoperative incisional pain at rest and in motion between the groups

**Figure 2:**
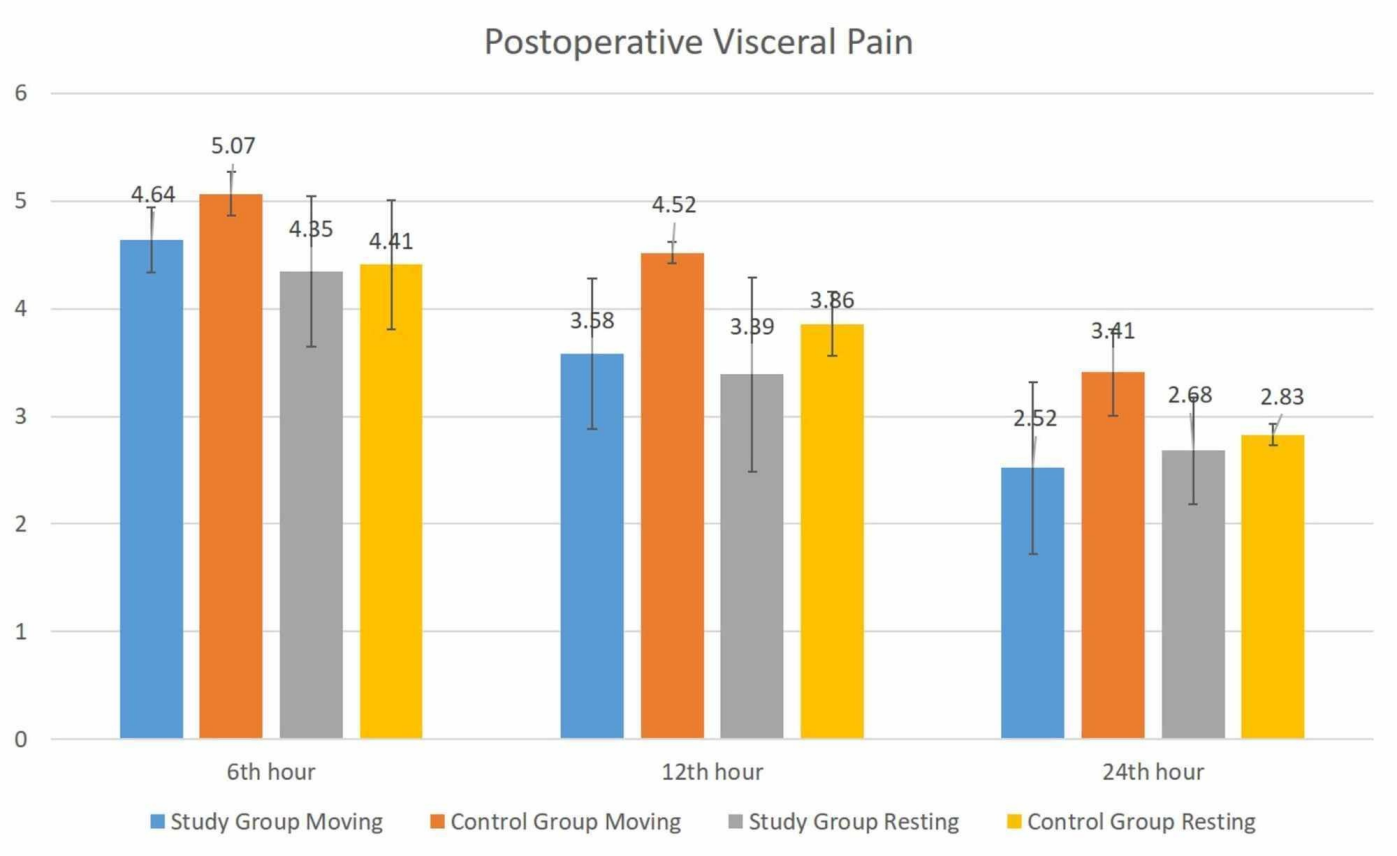
Postoperative visceral pain at rest and in motion between the groups

No statistically significant difference was observed between the groups in terms of visceral pain at rest at postoperative 6th, 12th and 24th hours. There was no statistically significant difference between both groups in terms of visceral pain in motion at the postoperative 6th hour (p > 0.05). Although there was a difference between the groups in terms of visceral pain in motion at the postoperative 12th and 24th hours, this was not statistically significant (p > 0.05). Although not statistically significant, remarkably visceral pain in motion was lower in the study group at the postoperative 12th and 24th hours. Distributions of incisional and visceral pain scores between the groups at rest and in motion are given in Figure 1 and Figure 2.

Although the need for additional analgesic was lower in the study group (25.8%) compared to the control group (44.8%), the difference between the groups was not statistically significant (p > 0.05).

No statistically significant difference was found between the groups in terms of the presence of nausea at postoperative 0-6 hours and postoperative 6-24 hours (p > 0.05). Again, no statistically significant difference was found between the groups in terms of the presence of vomiting at postoperative 0-6 hours and postoperative 6-24 hours (p > 0.05).

The need for additional anti-emetic medication was lower in the study group (19.4%) compared to the control group (37.9%), although the difference was not statistically significant (p > 0.05).

No statistically significant difference was found between the groups in terms of pain evaluated at postoperative 1st, 2nd, 3rd, 4th, 5th, 6th and 7th days (p > 0.05). Scoring in this evaluation was carried out by sum of the incisional and visceral pain scores at rest and in motion.

## Discussion

Glucocorticoids regulate humoral mediators triggered by trauma and modify postoperative physiologic, inflammatory, humoral and immunologic responses [5, 6]. C-reactive protein (CRP) is thought to play an important role in response to the body against trauma and inflammation. As is known, glucocorticoids exert their analgesic effect through the inhibition of phospholipase enzyme, blockage of cyclooxygenase and lipoxygenase pathways in inflammatory chain reaction, reduction of tissue bradykinin levels and release of neuropeptides from nerve ends [8-10].

In the present study, no statistically significant difference was observed between the groups in terms of incisional pain at rest at postoperative 6th, 12th and 24th hours (p > 0.05). Again no statistically significant difference was observed between the groups in terms of visceral pain at rest at postoperative 6th, 12th and 24th hours (p > 0.05).

There was no significant difference between the groups in terms of incisional pain at rest, namely when the patient was mobilized at the postoperative 6th, 12th and 24th hours (p > 0.05). There was no statistically significant difference between both groups in terms of visceral pain in motion at the postoperative 6th hour (p > 0.05). Although there was a difference between the groups in terms of visceral pain in motion at the postoperative 12th and 24th hours, this was not statistically significant (p > 0.05). Although not statistically significant, remarkably visceral pain in motion was lower in the study group at the postoperative 12th and 24th hours.

There was no statistically significant difference between the groups in terms of total pain obtained by sum of incisional and visceral pain scores from postoperative 1st day through 7th day (p > 0.05).

Postoperative 24-hour period mostly involves hospitalization. Although no significant difference was found between the groups in terms of the need for additional analgesic support other than anesthesia protocol applied in all patients (p > 0.05), the need for additional analgesic was markedly lower in the study group (25.8%) compared to the control group (44.8%).

In a study by McKenzie et al., perioperative single dose dexamethasone was found to decrease pain following abdominal surgery [11]. In another study by Holte and Kehlet, randomized controlled studies including many minor and major surgical procedures were analyzed in order to evaluate the effects of perioperative single dose glucocorticoids. In conclusion, it was found that glucocorticoids have highly limited or no effect on pain in major surgical procedures, while their effects increase in minor surgical procedures and especially in dental interventions [12].

Although it is not fully understood how antiemetic effects of glucocorticoids occur, they are thought to affect centrally through the inhibition of prostaglandin synthesis or the inhibition of endogenous opioids release [13].

Glucocorticoids bind to intracellular glucocorticoid receptors and exert their effects through protein synthesis and gene transcription [14]. Therefore, the biological effect begins within 1-2 hours. Unfortunately, in many studies glucocorticoids are given immediately before anesthesia induction. This also applies for studies with laparoscopic cholecystectomy [15].

Because the activation of early mediators of the metabolic response occurring against surgery begins with skin incision, it is crucial to administer glucocorticoids 1-2 hours before the operation in order to obtain desired effects postoperatively. In the present study, dexamethasone was preoperatively administered 90 minutes before skin incision.

In the current study, nausea and vomiting were evaluated as two periods. Looking at the obtained data, no statistically significant difference was found between the groups in terms of the presence and severity of nausea at 0-6 hours and 6-24 hours intervals (p > 0.05).

Again no statistically significant difference was found between the groups in terms of the presence and severity of vomiting at 0-6 hours and 6-24 hours intervals (p > 0.05).

Although the need for additional antiemetic drugs was lower in the study group (19.4%) compared to the control group (37.9%), the difference was not statistically significant.

In a meta-analysis, 17 placebo-controlled studies were reviewed, and it was demonstrated that a combination of 5-HT3 receptor antagonist and single dose dexamethasone can decrease postoperative nausea and vomiting. However, as a result of these studies there are still questions that have not been elucidated about optimal dose of the combination and necessity of 5-HT3 receptor antagonist [15, 16].

In a study by Aouad et al., preoperative 0.5 mg/kg dexamethasone was given to 110 children aged between 2-12 years who were scheduled for adenotonsillectomy operation, and it was found that postoperative nausea and vomiting decreased by 50% compared to placebo group [17].

In a study by Wang et al., dexamethasone administered at doses of 10 and 5 mg in 180 patients after cesarean section decreased postoperative nausea and vomiting, and the need for additional antiemetic drugs [18]. In another study, the preoperative use of dexamethasone in women undergoing emergency laparoscopic surgery decreased postoperative nausea and vomiting [19].

## Conclusions

In this study, when studies on several parameters including pain, nausea and vomiting were statistically analyzed, although no significant, the need for analgesics and anti-emetic drugs was lower in the study group which received dexamethasone compared to the control group.

We can expect better results with the use of multimodal analgesic and anti-emetic combination instead of a single agent in studies to be performed about the prevention of postoperative pain, nausea and vomiting.
